# Human serum albumin-based nanoparticles alter raloxifene administration and improve bioavailability

**DOI:** 10.1080/10717544.2022.2111479

**Published:** 2022-08-16

**Authors:** Shu-Jyuan Yang, Chih-Hao Chang, Tai-Horng Young, Chung-Hao Wang, Tzu-Hao Tseng, Man-Ling Wang

**Affiliations:** a Institute of Biomedical Engineering, College of Medicine and College of Engineering, National Taiwan University, Taipei, Taiwan; b Department of Orthopedics, National Taiwan University Hospital Jin-Shan Branch, New Taipei City, Taiwan; c Department of Orthopedics, National Taiwan University Hospital and National Taiwan University College of Medicine, Taipei, Taiwan; d CYBER ELITE LIMITED, Vistra Corporate Services Centre, Apia, Samoa; e Department of Orthopaedic Surgery, National Taiwan University Hospital, Taipei, Taiwan; f Graduate Institute of Clinical Medicine, National Taiwan University College of Medicine, Taipei, Taiwan; g Department of Anesthesiology, National Taiwan University Hospital and National Taiwan University College of Medicine, Taipei, Taiwan

**Keywords:** Osteoporosis, raloxifene, human serum albumin, poly(sodium styrene sulfonate), nanoparticle

## Abstract

Osteoporosis is a disease that reduces bone mass and microarchitecture, which makes bones fragile. Postmenopausal osteoporosis occurs due to estrogen deficiency. Raloxifene is a selective estrogen receptor modulator used to treat postmenopausal osteoporosis. However, it has a low bioavailability, which requires long-term, high-dose raloxifene administration to be effective and causes several side effects. Herein, raloxifene was encapsulated in human serum albumin (HSA)-based nanoparticles (Ral/HSA/PSS NPs) as an intravenous-injection pharmaceutical formulation to increase its bioavailability and reduce the treatment dosage and time. *In vitro* results indicated that raloxifene molecules were well distributed in HSA-based nanoparticles as an amorphous state, and the resulting raloxifene formulation was stabile during long-term storage duration. The Ral/HSA/PSS NPs were both biocompatible and hemocompatible with a decreased cytotoxicity of high-dose raloxifene. Moreover, the intravenous administration of the prepared Ral/HSA/PSS NPs to rats improved raloxifene bioavailability and improved its half-life in plasma. These raloxifene-loaded nanoparticles may be a potential nanomedicine candidate for treating postmenopausal osteoporosis with lower raloxifene dosages.

## 1. Introduction

With the advances in medicine and an aging population, aging-related diseases have gained attention, including osteoporosis. In osteoporosis, a reduction in bone mass and microarchitecture makes the bones fragile. The lack of obvious symptoms in osteoporosis increases the risk of fracture and may lead to dysfunction that affects quality of life and increases the risk of death (Ayub et al., 2021). Osteoporosis is a multifactorial disease with a complex pathophysiology that can be caused by age, like postmenopausal osteoporosis and aging osteoporosis, as well as disease or lifestyle habits, such as dysfunction, the long-term use of certain drugs, malnutrition, or bad habits (Tabatabaei-Malazy et al., 2017; Eskandarynasab et al., 2020). There are two groups of pharmacological osteoporosis treatments: antiresorptive and anabolic agents. Antiresorptive agents mainly reduce the bone resorption by inhibiting osteoclast activity while anabolic agents can induce new bone formation by stimulating osteoblasts (Tabatabaei-Malazy et al., 2017). Postmenopausal women have an estrogen deficiency that leads to increased bone absorption and accelerated bone loss by increasing osteoclast number and activity (Riggs, 2000), which increases the risk of bone fractures, especially in the spine, hip, and wrists. Postmenopausal osteoporosis treatment includes calcitonin, bisphosphonates, and active vitamin D_3_. While estrogen and anabolic steroids, also called hormone replacement therapy, are effective, increased risks of cancer and deep vein thrombosis prevents their use as first line therapies (Hochner-Celnikier, 1999). Selective estrogen receptor modulators (SERMs) can act on osteoblast estrogen receptors to reduce bone loss and increase bone density by activating and/or blocking estrogen pathways without negatively affecting the breast and endometrium (Riggs & Hartmann, 2003).

Raloxifene hydrochloride is a popular second-generation SERM that was approved by the US Food and Drug Administration in 1997 for the treatment and prevention of bone absorption caused by postmenopausal osteoporosis (Riggs & Hartmann, 2003; Saini et al., 2015). Raloxifene at the dosage of 60 mg must be taken once a day continuously for more than two years to significantly increase bone density and reduce the occurrence of fractures (Cooper et al., 2012). Raloxifene also lowers total and low-density lipoprotein cholesterol without increasing triglycerides or altering high-density lipoprotein cholesterol. Furthermore, preclinical data show that raloxifene also can display as an estrogen antagonist to reduce the risk of breast cancer incidence in postmenopausal women (Delmas et al., 2002; Waters et al., 2012). Because raloxifene is hydrophobic molecule and has a low aqueous solubility, it is primarily administered orally as tablets. However, the oral administration of raloxifene undergoes extensive intestinal glucuronidation and phase II metabolism in the liver (the first-pass effect), which reduce its bioavailability to 2% (Jagadish et al., 2010; Ahmed & Badr-Eldin, 2018). Additionally, the long-term, high-dose administration of raloxifene can cause several side effects, such as hot flashes, nausea, deep vein thrombosis, stroke, leg cramps, swelling, and cold-like symptoms, which results in poor patient compliance (Modi et al., 2016).

Nanomedicine, the medical application of nanotechnology, has many advantages over conventional pharmaceuticals. These advantages include the improved drug bioavailability; the increased therapeutic efficiency of lower doses at a lower frequency; the targeting of specific tissues; a prolonged shelf-life; and the increased patient compliance and convenience (Orive et al., 2003). Therefore, previous studies have loaded raloxifene in lecithin-chitosan based nanoparticles (Murthy et al., 2020), lipid carriers (Murthy et al., 2020), or co-loaded it with vitamin D in D-α-tocopherol polyethylene glycol 1000 succinate nanoparticles (Ahmed & Badr-Eldin, 2018) to improve its bioavailability.

Human serum albumin (HSA) is one of the most abundant endogenous proteins in human blood. Many metabolic compounds and therapeutic drugs can be bound to HSA and transported by HSA in the blood (Peters, 1958), which indicates that HSA can be used as a natural drug carrier. HSA is often exploited as a carrier for hydrophobic chemotherapeutics for many years. Abraxane® is the first one of HSA-based nanocarriers to attract attention, which is facilitated by non-covalent bonding of paclitaxel with HSA hydrophobic functional groups (Kouchakzadeh et al., 2015). HSA also increases the compatibility and circulation half-life of nanocarriers in the blood (Tirkey et al., 2017; Lee et al., 2018). In order to provide good blood compatibility and anticoagulant activity in drug carriers, the sulfate group-containing polymers such as poly(sodium 4-styrenesulfonate) (PSS) can be used (Li et al., 2013). PSS is a synthetic and frequently investigated polyelectrolyte that dissociates in aqueous solutions to bear negative sulfate groups (Fuenzalida et al., 2014). PSS has been applied to duplicate the anionic sulfate domains of heparin to synthesize heparin-mimicking polymers with anticoagulant activity (Huang et al., 2014). Moreover, it has been untiled in the multilayer film development led to a very good biocompatibility for endothelial cells, osteoblast-like cells and chondrosarcoma cells (Boura et al., 2003).

In this study, we encapsulated raloxifene in HSA-based nanoparticles as an intravenous (IV)-injection formulation. We hypothesized that using HSA and PSS as carriers of the hydrophobic drug raloxifene may improve the blood compatibility and circulation half-life of nanocarriers as well as improve raloxifene bioavailability, which reduces the required dosage.

## 2. Experimental section

### 2.1. Materials

HSA, (≥96%); raloxifene (Ral); poly(sodium 4-styrenesulfonate) (PSS, MW = 70,000 Da); ferric chloride (97%); 1,10-phenanthroline (≥99%); 3-(4,5-dimethylthiazol-2-yl)-2,5-diphenyltetrazolium bromide (MTT); fluorescein 5(6)-isothiocyanate (FITC); and dimethyl sulfoxide (DMSO) were purchased from Sigma-Aldrich (USA). EVISTA® (raloxifene) 60 mg film-coated tablets were obtained from Eli Lilly and Company (USA).

### 2.2. Ral/HSA/PSS nanoparticle preparation

The Ral/HSA/PSS nanoparticle (NP) batches were developed via modifying the lyophilization-hydration method (Peng et al., 2008; Tsai et al., 2017). Briefly, the preparation process is as follows: the raloxifene powder was dissolved in DMSO at the concentration of 0.5 mg/mL, and the HSA-PSS mixture was dripped slowly at the Ral/HSA/PSS weight ratio of 1/2/8, 1/2/10, or 1/2/12. After lyophilization, the Ral/HSA/PSS mixture was then mixed in 1 mL of double-distilled water (ddH_2_O) and ultrasonicated to form Ral/HSA/PSS NPs. The prepared nanocarrier solutions were stored until use.

### 2.3. Ral/HSA/PSS nanoparticle characterization

#### 2.3.1. Particle characteristics

The hydrodynamic size and zeta potential of the prepared Ral/HSA/PSS NPs were measured using a Zetasizer Nano-ZS90 (Malvern Instruments Ltd, UK). The dried, carbon-coated copper grids with Ral/HSA/PSS NP precipitates were examined under a transmission electron microscope (TEM, Hitachi H-7500, Tokyo, Japan) to observe the relationship between nanoparticle surface morphology and different PSS content.

#### 2.3.2. Determination of protein configuration change

The absorbance and fluorescence spectra of a FITC-HSA conjugate/PSS mixture in ddH_2_O and 89% (w/w) DMSO solution were recorded on an ELISA microplate reader (SpectraMax M2 Multi-Mode Microplate Reader, Molecular Devices, USA). The FTIR spectra of HSA, HSA/PSS mixture and Ral/HSA/PSS NPs were determined by a Nicolet™ Summit FTIR Spectrometer (Thermo Fisher Scientific, MA, USA) equipped with MicromATR.

#### 2.3.3. Encapsulation efficacy and drug distribution analysis

The encapsulation efficiency of raloxifene in the prepared Ral/HSA/PSS NPs was determined using the spectrophotometric method (Mathrusri-Annapurn et al., 2007). After re-lyophilization and re-suspension in DMSO, the Ral/HSA/PSS NP solutions were filtered through a 0.22-µm Millex GP filter unit (Merck Millipore Ltd., Tullagreen, Ireland) to remove the NPs, and 50 µL of the filtered solution was incubated with 0.5 mL of 0.03 M ferric chloride solution and 0.75 mL of 0.01 M 1,10-phenanthroline solution for 15 min at 60 °C. The absorbance of the blood-red precipitate was measured at 510 nm using the ELISA microplate reader (SpectraMax M2 Multi-Mode Microplate Reader, Molecular Devices, USA). Raloxifene powder was dissolved in DMSO and serially diluted into raloxifene standard solutions to create a 12.5 − 200 μg/mL calibration curve. The raloxifene encapsulation efficacy (EE) and drug-loading content (DC) of raloxifene in the prepared nanoparticles were calculated with the following equations, respectively:

$$\begin{array}{l} \text{EE }\left(\%\right)=\left(\text{Weight of raloxifene in the particles}/\text{Total weight of feeding raloxifene}\right)\times \text{1}00,\text{ and}\\ \text{DC }\left(\%\right)=\left(\text{Weight of raloxifene in the particles}/\text{Total weight of particles}\right)\times \text{1}00. \end{array}$$

Moreover, powder X-ray diffraction (XRD) patterns of raloxifene, HSA, PSS, and Ral/HSA/PSS NPs were investigated by a D2 PHASER X-ray diffractometer (Bruker AXS Inc., WI, USA) equipped with Cu Kα radiation (λ = 1.5418 Å).

#### 2.3.4. Stability analysis

The stability of the prepared Ral/HSA/PSS NPs in different solutions was assessed. For this purpose, the Ral/HSA/PSS NPs were suspended in 1 mL ddH_2_O or Gitose injection solution (5% dextrose solution, Nang Kuang Pharmaceutical Co. Ltd., Taiwan) at 4 °C. The changes of particle size were determined by a Zetasizer Nano-ZS90 at 0, 1, 4, 7, 11, 18, 21, 25, and 28 days post-preparation. The stability of the particles in Dulbecco’s Modified Eagle Medium (DMEM, Gibco) supplemented with or without 10% (v/v) fetal bovine serum (FBS) at 4 °C was also evaluated.

Moreover, the storage stability of the nanoparticles is a critical factor that must be considered during formulation design and development (Gradauer et al., 2013). Herein, the Ral/HSA/PSS NPs were re-lyophilized and preserved at room temperature. The re-lyophilized Ral/HSA/PSS NPs were then re-suspended in 1 mL ddH_2_O and the particle size measured with a Zetasizer Nano-ZS90 at 0, 1, 3, 5, 7, 10, 14, 21, and 28 days post-lyophilization.

### 2.4. In vitro drug-release assay

The drug-release profiles of raloxifene from the Ral/HSA/PSS NPs were investigated using the dialysis-bag diffusion method (Yang et al., 2020). Briefly, 2 mL of the prepared Ral/HSA/PSS NPs in phosphate buffered saline (PBS) were loaded into a 3.5 kDa molecular weight cutoff CelluSep dialysis membrane (Membrane Filtration Products, Seguin, TX, USA). The dialysis membrane was sequentially soaked in 28 mL of PBS with or without 10% FBS at pH 7.4. This drug released system was incubated at 37 °C with 150 rpm shaking for 2 days. At 1, 2, 3, 4, 6, 8, 10, 23, 30, and 48 hours, 0.25 mL of the buffer was removed to measure raloxifene using the spectrophotometric method.

### 2.5. In vitro cytotoxicity assay

The Hs-68 cell line (human newborn normal diploid fibroblasts) was cultivated in DMEM complemented with 10% FBS and 1% Gibco® antibiotic-antimycotic solution (Thermo Fisher Scientific, USA). A human osteosarcoma cell line, MG-63, was cultivated in MEM supplemented with 10% FBS and 1% Gibco® antibiotic-antimycotic solution. Both cell lines were cultured at 37 °C in a 5% CO_2_ atmosphere, and the culture media changed on alternate days until the cells reached confluence.

Hs-68 or MG-63 cells were separately seeded into 96-well plates at a density of 5 × 10^3^ cells/well, respectively. After 24 h incubation, the cells were then incubated in fresh media supplemented with either Ral/HSA/PSS NPs or free raloxifene at the raloxifene concentrations of 0, 1, 10, 25, and 50 μg/mL. After 24 or 72 h, the cells were placed in fresh media containing MTT reagents and incubated for another 3 h (Yang et al, 2020). Finally, the absorbance was measured at 570 nm with a UV/Vis spectrophotometer/microplate reader (SpectraMax Plus, Molecular Devices, USA). The cell viability of each group was calculated and expressed as a percentage relative to the control group.

### 2.6. Hemocompatibility assay

For the hemocompatibility assay, heparin-stabilized blood samples were collected from Wistar rats, and the whole blood was diluted in PBS and washed several times at 1000 rpm for 5 min to remove blood plasma. Each 200 μL blood sample was treated with 200 μL of Ral/HSA/PSS NPs ([Raloxifene] = 10, 25, 50, 75, 100, 200, 300, 400, of 500 μg/mL), PBS (NC, negative control), and ddH_2_O (PC, positive control). The samples were mixed at 37 °C and a stirring rate of 100 rpm for 1 h, and then centrifuged at 4000 rpm for 5 min. The absorbance of the supernatant was recorded with an ELISA microplate reader at 540 nm. The hemolysis percentage of the samples was calculated using the following equation (Chauhan et al., 2019):

Hemolysis percentage (%) = [(Absorbance of test samples − Absorbance of negative control)/(Absorbance of positive control − Absorbance of negative control)] × 100.

Hemolysis percentage (%)=[(Absorbance of test samples − Absorbance of negative control)/(Absorbance of positive control − Absorbance of negative control)]× 100.

### 2.7. Pharmacokinetic analysis

The pharmacokinetic analysis was conducted by determining the raloxifene concentration in plasma. Female Wistar rats (180–200 g) were purchased from the National Laboratory Animal Center (Taipei, Taiwan), and all experimental rats received care on the basis of the Guide for the Care and Use of Laboratory Animals (8th edition). The animal experiments were applied in accordance with protocols approved by the National Taiwan University College of Medicine and College of Public Health Institutional Animal Care and Use Committee.

The animals were divided into two equal groups (*n* = 5). One group received an EVISTA suspension (30 mg of raloxifene/kg) by oral gavage, and the other group received the Ral/HSA/PSS NPs (5 mg of raloxifene/kg) via IV injection. Whole blood samples (0.15 mL) were collected from the tail vein in blood collection tubes containing heparin pre-dose and at 1, 2, 3, 4, 6, 8, and 24 h. Plasma was separated from the blood components by centrifugation at 1000 ×g at 4 °C for 10 min. A validated high-performance liquid chromatography method was employed to analyze the samples after suitable modifications (Murthy et al., 2020).

### 2.8. Statistical analysis

All data were presented as the mean ± standard deviation. The student’s t-test was used to analyze the significance of differences between each group. A p value less than 0.05 was considered statistically significant.

## 3. Results and discussion

### 3.1. Characterization of ral/HSA/PSS NPs

**Figure 1.**
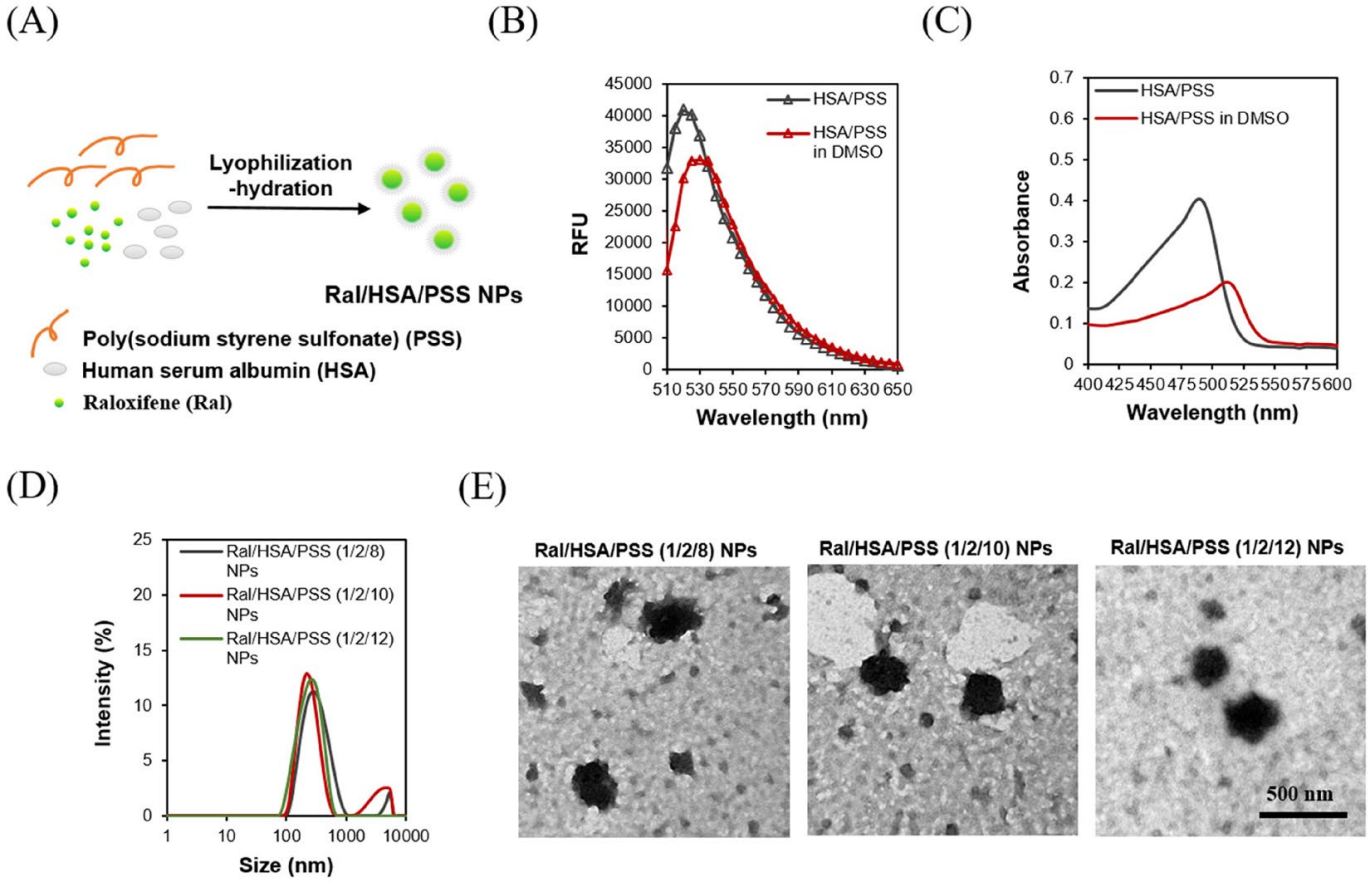
(A) The Ral/HSA/PSS NPs were prepared by the lyophilization-hydration method. (B) Fluorescence spectrum of FITC-HSA/PSS mixture in ddH_2_O (black) or 89% DMSO (red) solution (λ_exc_ = 488 nm). (C) Absorption spectrum of FITC-HSA/PSS mixture in ddH_2_O (black) or 89% DMSO (red). (D) Particle size distribution of Ral/HSA/PSS NPs at three formulation ratios. (E) TEM images of Ral/HSA/PSS NPs. Samples were negatively stained with 2% uranyl acetate before imaging. Scale bar = 500 nm.

The solubility of raloxifene in water is low (about 0.56 mg/L), which makes it difficult to administer intravenously. Unfortunately, the extensive intestinal glucuronidation and first-pass metabolism of oral raloxifene results in a low bioavailability (2%) (Jagadish et al., 2010), which increases the cost of osteoporosis treatment and its side effects like venous thrombosis (Modi et al., 2016). To increase bioavailability, these intravenously injectable raloxifene-loaded nanoparticles (Ral/HSA/PSS NPs) were exploited by the lyophilization-hydration method (Figure 1A).

Raloxifene may be attached by a non-covalent bond with HSA hydrophobic functional groups, like the Abraxane® nanocarrier (Kouchakzadeh et al., 2015). To characterize the conformation change of HSA in the preparing Ral/HSA/PSS NPs, FITC-HSA/PSS mixture was exposed to DMSO and the resulting fluorescence behavior was measured (Figure 1B). The fluorescence intensity of FITC-HSA/PSS mixture decreased with in the presence of DMSO by a 10 nm redshift of the emission maximum, which indicates that DMSO may alter the HSA conformation. Data from Samanta et al. in 2013 indicated that increasing DMSO in an aqueous solution of HSA also increased the hydrophobicity of the tryptophan microenvironment (Pabbathi et al., 2013). The absorption spectrum of FITC-HSA/PSS mixture was significantly affected by the solvents (Figure 1C). The FITC-HSA/PSS mixture might be well-dispersed in ddH_2_O but aggregate in 89% (w/w) DMSO, as indicated by a redshift peak in absorption spectra (Collison et al., 2001; Traiphol & Charoenthai, 2008). Therefore, these non-polar aromatic amino acids might become exposed through DMSO-induced protein conformational change, which would enable their interaction with the hydrophobic raloxifene and cause aggregates in DMSO.

The particle size of Ral/HSA/PSS NPs correlated with the PSS-incorporated content (Figure 1D), which indicates that when the ratio of Ral/HSA/PSS increased from 1/2/8 to 1/2/10, the particle size decreased from 302.8 nm to 266.9 nm. However, additional PSS-incorporated content in Ral/HSA/PSS NPs (Ral/HSA/PSS = 1/2/12) increased the particle size (269.2 nm) and size distribution (PdI = 0.467). Moreover, the zeta potential of Ral/HSA/PSS NPs was positively dependent on the PSS-incorporated content, decreasing from −44.4 mV to −48.1 mV when the ratio of Ral/HSA/PSS increased from 1/2/8 to 1/2/12. The LD and DC of raloxifene in NPs prepared at the Ral/HSA/PSS ratio of 1/2/10 were 99.4% and 8.3%, respectively. TEM images demonstrated that the surface morphology of Ral/HSA/PSS NPs was rough and independent of Ral/HSA/PSS ratio (Figure 1E). The Ral/HSA/PSS NPs prepared at the Ral/HSA/PSS ratio of 1/2/10 displayed an optimal particle size and size distribution, so we decided to prepare Ral/HSA/PSS NPs at 1/2/10 ratio for the remaining experiments.

**Figure 2.**
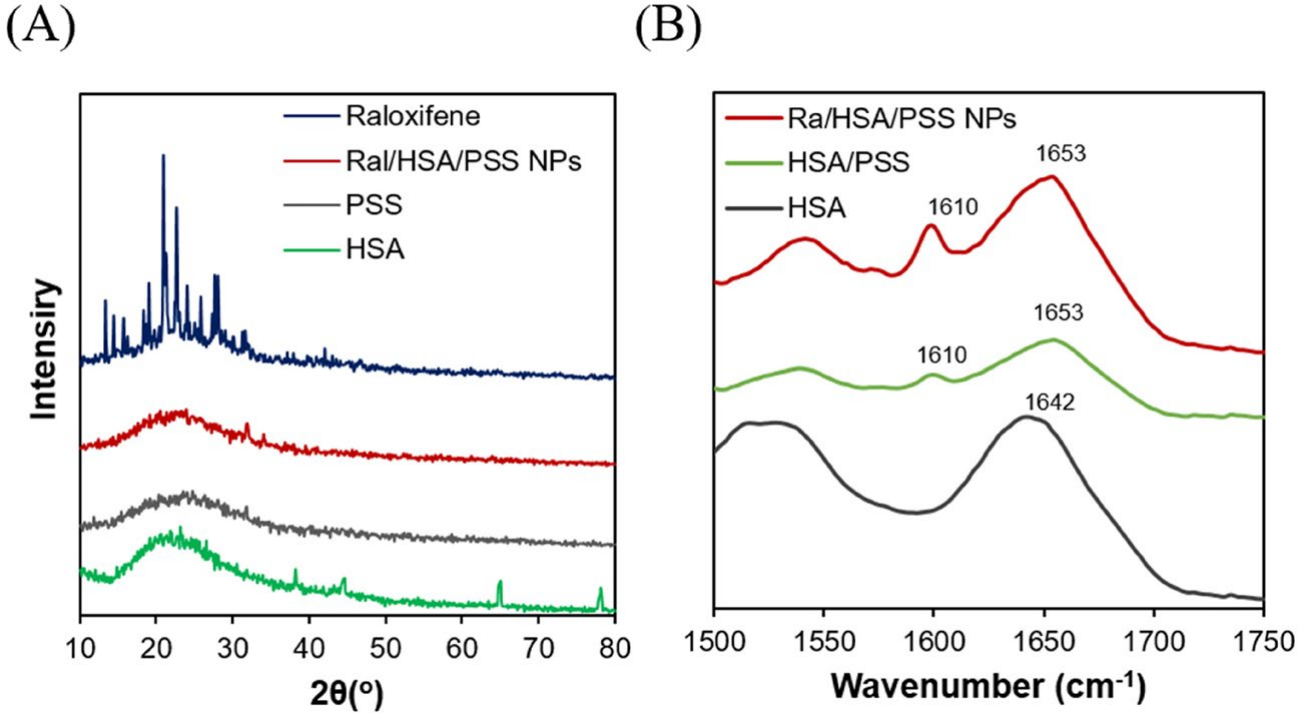
(A) XRD diagrams of raloxifene (blue), PSS (gray), HSA (green), and Ral/HSA/PSS NPs (red). (B) FTIR spectra of HSA (black), HSA/PSS (green) mixture, and Ral/HSA/PSS NPs (red).

Because raloxifene is hydrophobic and a crystalline molecule, the XRD pattern could determine the state of raloxifene in Ral/HSA/PSS NPs. The diffraction pattern of raloxifene contained sharp peaks appearing at 14 − 32 degrees (2θ) that indicated its strong crystallization but disappeared in the Ral/HSA/PSS NPs diffraction pattern (Figure 2A). These results indicate that the encapsulation of raloxifene in HSA-PSS-based nanoparticles was successful; the raloxifene molecules displayed either in an amorphous state or small crystals well-dispersed in the prepared Ral/HSA/PSS NPs. Both this amorphous state and small crystals could be advantageous for raloxifene molecules to obtain an advanced dissolution rate and a better bioavailability in further clinical applications (Hu et al., 2015).

We next investigated the FTIR spectra of HSA, HSA-PSS mixture, and Ral/HSA/PSS NPs (Figure 2B). The characteristic peak at 1642 cm^−1^ in the HSA spectrum was assigned random coils; it disappeared in the HSA-PSS and Ral/HSA/PSS NPs spectra and two peaks at 1632 and 1610 cm^−1^ were identified, which were mainly associated with α-helical structures and intermolecular β-sheet structures, respectively (Lu et al., 2015). Moreover, the intensities of the two secondary-structure absorption peaks in Ral/HSA/PSS NPs spectrum were higher than those in the HSA-PSS spectrum. These results indicate that PSS incorporation and raloxifene loading affect the HSA secondary structures.

**Figure 3.**
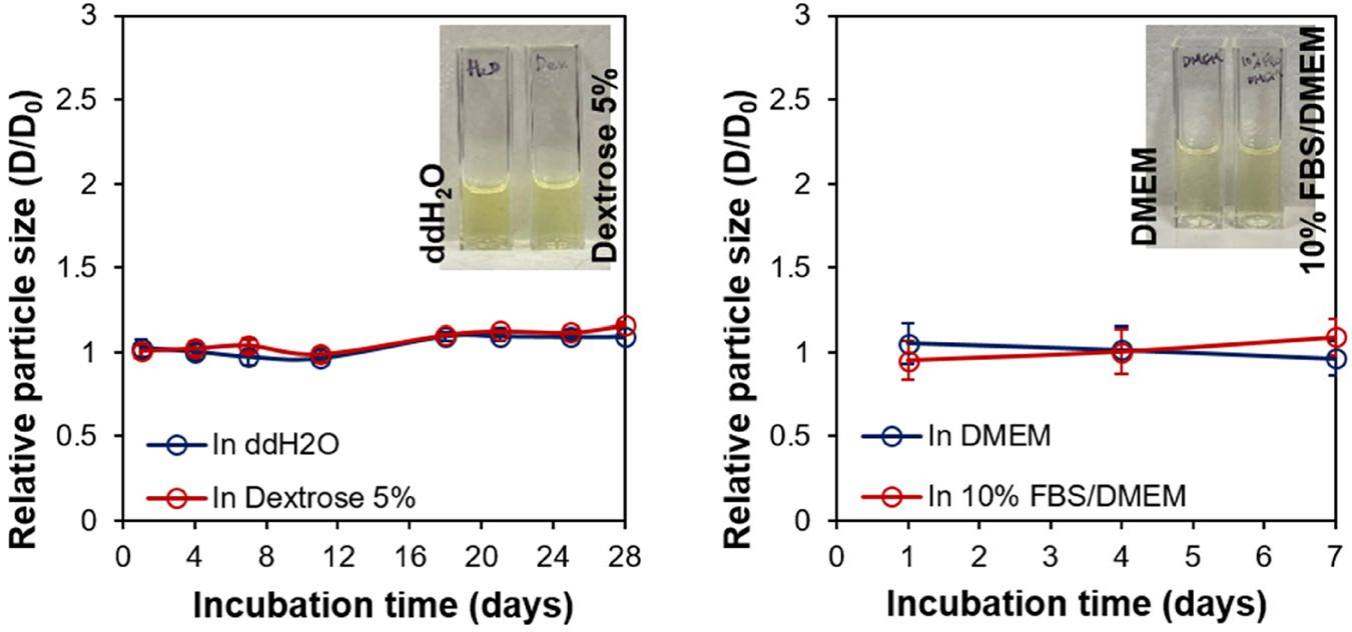
*Left:* Change in the relative particle size of Ral/HSA/PSS NPs in ddH_2_O (blue) or dextrose 5% (red) over 28 days. *Right:* Change in the relative particle size of Ral/HSA/PSS NPs in DMEM alone (blue) or supplemented with 10% FBS (red) over 1 week.

The colloidal stabilities of Ral/HSA/PSS NPs in ddH_2_O and Gitose injection 5% solution were assessed over a 4-week storage period at 4 °C. No significant particle size changes were observed in either ddH_2_O or Gitose injection 5% solution (Figure 3), which indicated a good colloidal stability of Ral/HSA/PSS NPs in both. The stability of Ral/HSA/PSS NPs in DMEM and 10% FBS supplemented DMEM were also investigated at 4 °C over a 1-week storage period. These results showed that the Ral/HSA/PSS NPs were stable in both media, without any undesired protein absorption that may cause severe NP aggregation in the blood (Yang et al., 2020).

**Figure 4.**
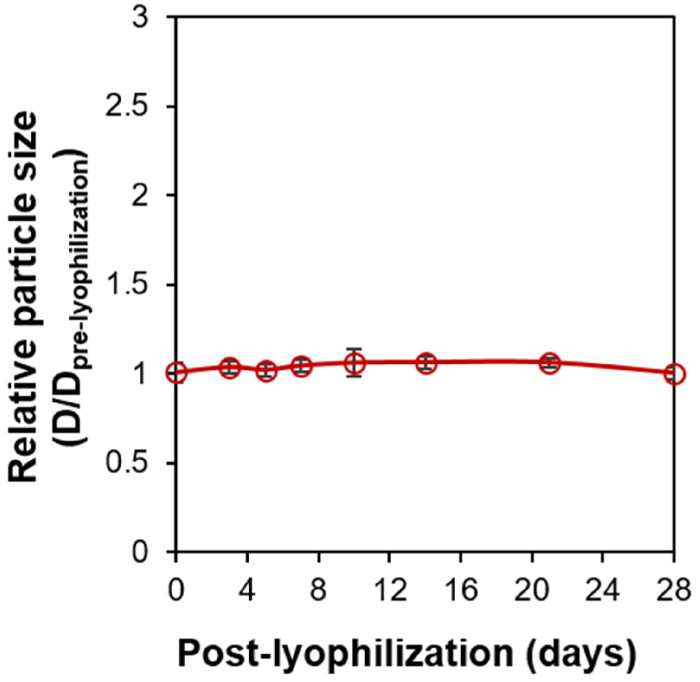
The storage stability of Ral/HSA/PSS NPs as measured by relative particle size after dissolving re-lyophilized Ral/HSA/PSS NPs in 1 mL ddH_2_O at 0, 1, 3, 5, 7, 10, 14, 21, and 28 days.

A good pharmaceutical preparation must have good storage stability and shelf-life, which are key factors that must be considered in the formulation design and development process. The storage stability of Ral/HSA/PSS NPs was determined by dissolving the re-lyophilized Ral/HSA/PSS NPs in 1 mL ddH_2_O and measuring the particle size change. No significant differences in particle size were discovered for Ral/HSA/PSS NPs, even after being re-lyophilized and stored at room temperature for 28 days (Figure 4). Our Ral/HSA/PSS NPs show an excellent stability during the formulation design and development process.

### 3.2. In vitro drug-release

**Figure 5.**
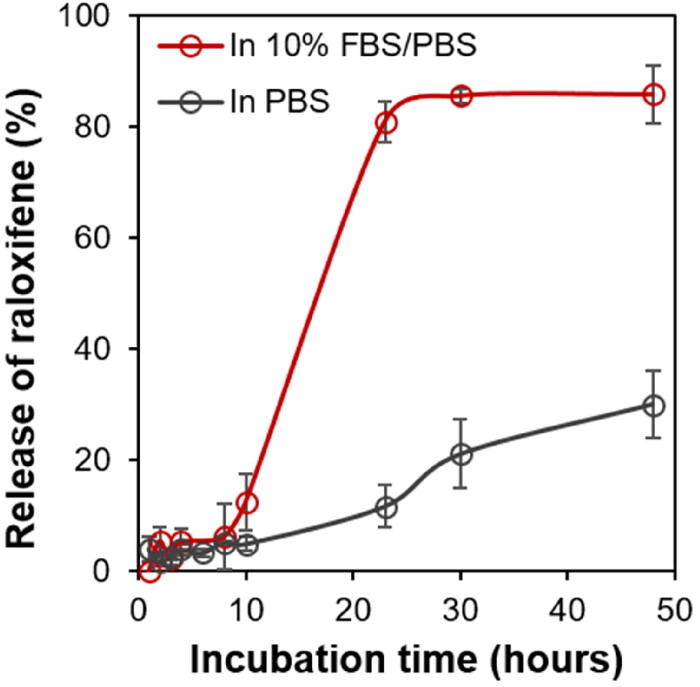
The percent release profiles of raloxifene from Ral/HSA/PSS NPs in PBS (black) and PBS supplemented with 10% FBS (red).

Raloxifene was encapsulated in the prepared Ral/HSA/PSS NPs to establish an IV medicine that avoids extensive intestinal glucuronidation and first-pass metabolism to enhance bioavailability. More than 95% of the bioavailable raloxifene administered orally binds to plasma proteins (Heringa, 2003). Therefore, to assess the effect of presence serum protein on raloxifene release, we performed the drug-release test at 37 °C in both PBS and PBS supplemented with 10% FBS. In PBS, only 30% of the raloxifene was released from Ral/HSA/PSS NPs at 48 h due to its low solubility in the aqueous solution (Figure 5). However, at 48 h, raloxifene release reached almost 85% in PBS with 10% FBS supplement. These results show that the raloxifene-loaded in Ral/has/PSS NPs is easily and continuously released to bind serum proteins, suggesting similar results in the blood following IV administration.

### 3.3. In vitro cytotoxicity and hemocompatibility studies

**Figure 6.**
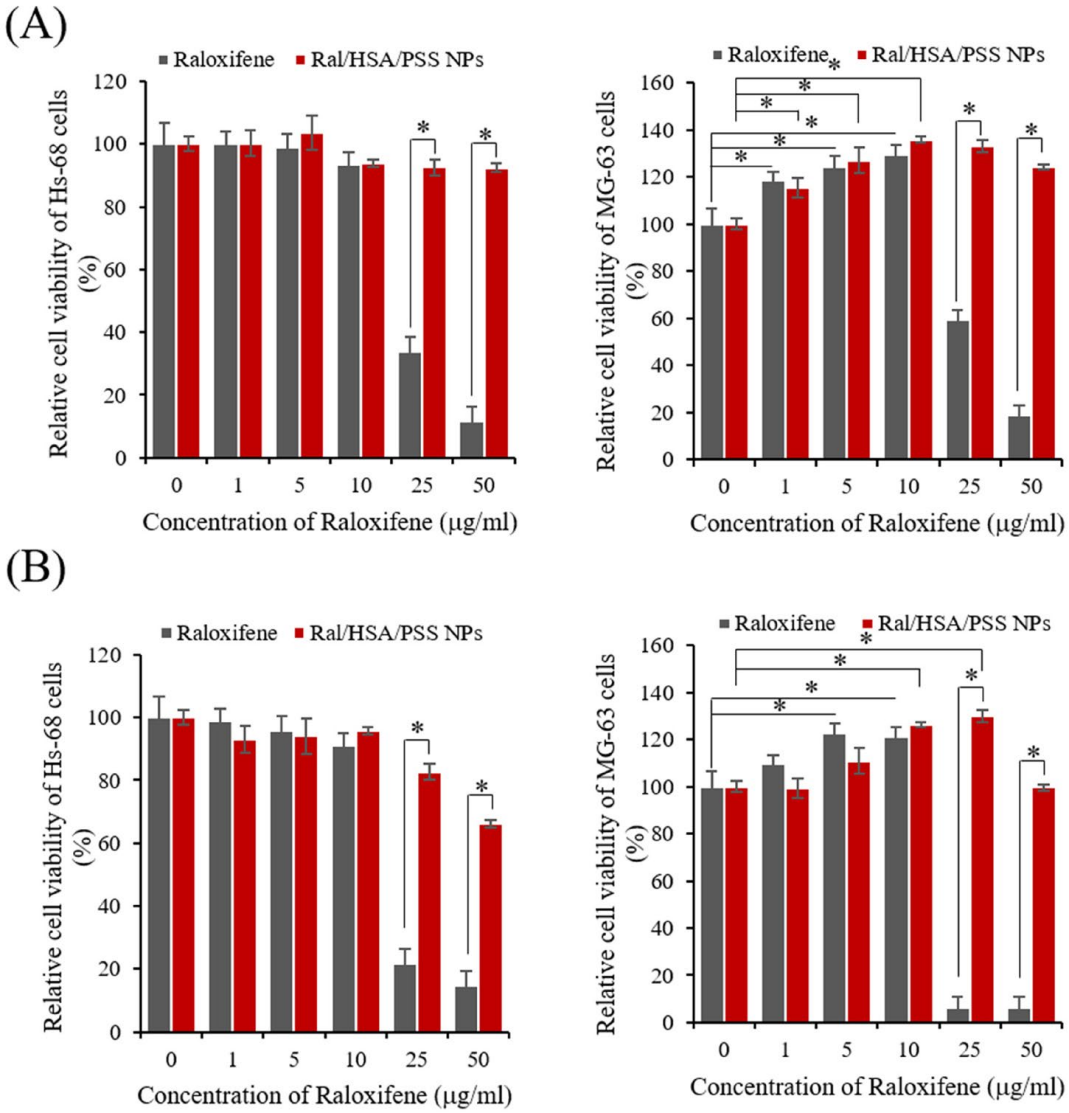
*In vitro* cytotoxicity of free raloxifene (black) and Ral/HSA/PSS NPs (red) in Hs-68 (left) and MG-63 (right) cells at (A) 24 h and (B) 72 h. *P < 0.05.

We first evaluated the cytotoxicity of free raloxifene and Ral/HSA/PSS NPs with raloxifene concentrations of 0, 1, 5, 10, 25, and 50 μg/mL on Hs-68 and MG-63 cells for 24 h. The free raloxifene and Ral/HSA/PSS NPs exhibited a dose-dependent cytotoxicity on Hs-68 cells; free raloxifene displayed severe cytotoxicity at concentrations above 25 µg/mL (Figure 6A). But the viability of Ral/HSA/PSS NPs-treated Hs-68 cells was always higher than 90% even at higher raloxifene concentration (50 μg/mL), indicating that the designed nanoparticles can reduce the toxicity of high dosages of raloxifene.

Moreover, the cell viability of MG-63 cells exposed to 25 μg/mL free raloxifene was 59% and decreased to 18% following exposure to 50 μg/mL raloxifene, which was similar to results with Hs-68 cells. However, low concentrations (≤10 µg/mL) of raloxifene significantly increased MG-63 cell viability in a dose-dependent manner, with the maximal viability (130%) observed at 10 µg/mL due to effects on osteoblast activity (Taranta et al., 2002; Muzio et al., 2013). Similar results were observed for Ral/HSA/PSS NPs-treated MG-63 cells, although the viability of Ral/HSA/PSS NPs-treated MG-63 cells remained above 100%, even at raloxifene concentrations greater than 50 μg/mL.

Moreover, the cytotoxicity of Hs-68 cells increased along with the incubation periods for free raloxifene and Ral/HSA/PSS NPs (Figure 6B). For MG-63 cells, the viability did not change with the treatment duration of free raloxifene at low concentrations (≤10 μg/mL) but decreased to 6% with extended durations of 25 µg/mL raloxifene treatment. However, the prepared Ral/HSA/PSS NPs effectively improved the osteoblast activity of MG-63 cells at 25 μg/mL raloxifene. Even at 50 μg/mL raloxifene, Ral/HSA/PSS NP-exposed MG-63 cells retained a viability similar to that of the control group, which indicates that the prepared Ral/HSA/PSS NPs are biocompatible and can significantly reduce the toxicity of the raloxifene itself.

**Figure 7.**
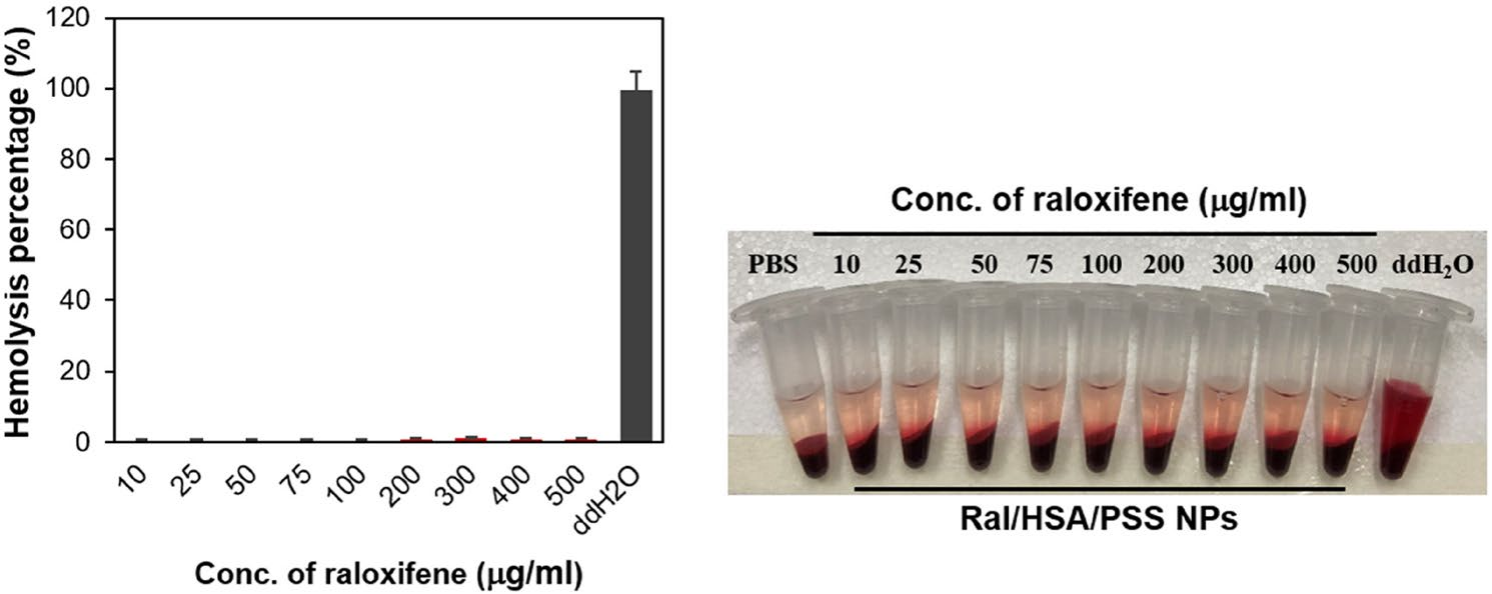
Hemocompatibility of Ral/HSA/PSS NPs.

The hemolysis test is another critical toxicity test that must be performed, especially for injectable nanomaterials (Chauhan et al., 2019). The hemolytic potential of Ral/HSA/PSS NPs was nearly equivalent to the negative control, PBS; even at concentrations five times the therapeutic dosage (100 µg/mL; Figure 7). Together, these results indicate that this designed HSA-based nanoparticle may reduce the toxicity of high raloxifene dosages to cells without causing hemolysis and might be a safe raloxifene delivery system for IV administration.

### 3.4. Pharmacokinetic analysis

**Figure 8.**
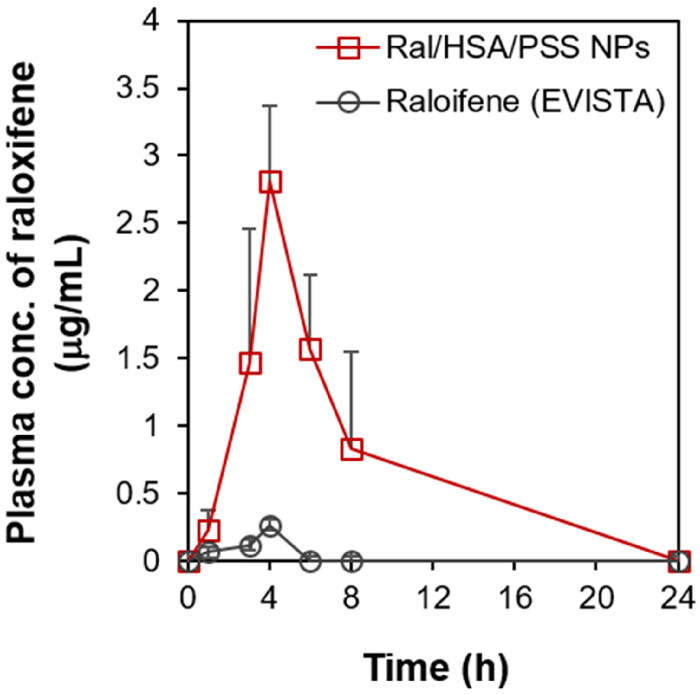
*In vivo* pharmacokinetic profile of raloxifene plasma levels following treatment with either EVISTA (black) or Ral/HSA/PSS NPs (red) in a female Wistar rat model. All data points are represented as mean ± SD (*n* = 5).

To improve the bioavailability and reduce the raloxifene dosage, we encapsulated raloxifene in HSA-based nanoparticles as an IV pharmaceutical formulation, as opposed to the current oral administration of raloxifene tablets. We next assessed the optimized Ral/HSA/PSS NP efficacy with IV pharmacokinetic (PK) studies in female Wistar rats. The oral administration of raloxifene (EVISTA) tablets at either 10 or 20 mg/kg resulted in no detectable plasma concentrations of raloxifene. Therefore, we increased the oral dose to 30 mg/kg to achieve adequate plasma detection of raloxifene. And there was no detectable raloxifene concentration in plasma after IV administration of Ral/HSA/PSS NPs with a raloxifene concentration of 2.5 mg/kg, but the IV administration of Ral/HSA/PSS NPs at a raloxifene concentration of 5 mg/kg could attain the sufficient plasma raloxifene concentration for detection by HPLC. Therefore, the final administrated raloxifene dose used to evaluate its pharmacokinetic parameters *in vivo* was mainly based on the raloxifene concentration in plasma that could be detected by HPLC. The PK profile of raloxifene in the EVISTA-treated group was remarkably distinct from that of the Ral/HSA/PSS NP-treated group (Figure 8). The maximum plasma concentration (C_max_) of raloxifene in the Ral/HSA/PSS NP-treated group was 10.8 times greater than that of the EVISTA-treated group at 4 h post-administration, even when the raloxifene dose in the Ral/HSA/PSS NP-treated group was only one-sixth of the EVISTA-treated group. The mean residence time (MRT) for EVISTA and Ral/HSA/PSS NPs were 3.32 and 5.89 h, respectively; the clearance parameter (CL/F) for each treated group was 44.78 and 0.29 L/h/kg, respectively (Table 1) (Hochner-Celnikier, 1999; Sinha et al., 2008). Moreover, the volume of distribution (Vd/F) of Ral/HSA/PSS NPs was 0.95 L/kg, and it was much lower than that of EVISTA (22.73 L/kg), reveling the IV administration of Ral/HSA/PSS NPs could avoid the rapid spread of raloxifene into the body tissue to decrease the risk of unfavorable effects (Tsai et al., 2017). These results indicate that these Ral/HSA/PSS NPs by IV administration could avoid the extensive intestinal glucuronidation and first-pass metabolism of raloxifene to increase its bioavailability, prolong its half-life in the body, and reduce the required dosage for postmenopausal osteoporosis treatment.

**Table 1.** Raloxifene pharmacokinetic parameters in female Wistar rats.

	EVISTA	Ral/HSA/PSS NPs
C_max_ (µg/mL)	0.26	2.81
T_max_ (h)	4	4
AUC_0-t_ ((ng·t/mL)/(mg/kg))*	22.33	3468.0
CL/F (L/kg·h)	44.78	0.29
Vd/F (L/kg)	22.73	0.95
MRT (h)	3.32	5.89

C_max_ = maximum plasma concentration, T_max_ = the time take to reach C_max_, AUC_0-t_ = area under the curve, MRT = mean residence time, CL = clearance, Vd = volume of distribution, F = bioavailability.

* Data normalized based on the dose (mg) and body weight (kg).

## 4. Conclusion

To convert raloxifene to an IV pharmaceutical formulation and increase its bioavailability, we encapsulated raloxifene in HSA-based nanoparticles (Ral/HSA/PSS NPs) with a lyophilization-hydration method to improve the compatibility and circulation half-life of raloxifene in the blood. *In vitro* results indicated that the raloxifene molecules were successfully encapsulated in HSA-based nanoparticles in an amorphous state and that this raloxifene formulation was stabile during long-term storage. Further *in vitro* experiments demonstrated that Ral/HSA/PSS NPs are biocompatible and hemocompatible as well as function to decrease the cytotoxicity of high raloxifene doses. Moreover, IV administration of the prepared Ral/HSA/PSS NPs to rats demonstrated improved bioavailability and a slow release of raloxifene into the plasma, which prolonged its half-life in the body and lowered the effective dosage and unfavorable effects. Therefore, this nanomedicine formulation may be a potential candidate for IV administration of raloxifene; although further evaluation is still ongoing, this raloxifene-loaded nanomedicine formulation might efficiently reduce bone loss and increase bone density to treat postmenopausal osteoporosis in the future.

## Data Availability

The data that support the findings of this study are available from the corresponding author, C.H. Chang, upon reasonable request.
